# Proton transport through nanoscale corrugations in two-dimensional crystals

**DOI:** 10.1038/s41586-023-06247-6

**Published:** 2023-08-23

**Authors:** O. J. Wahab, E. Daviddi, B. Xin, P. Z. Sun, E. Griffin, A. W. Colburn, D. Barry, M. Yagmurcukardes, F. M. Peeters, A. K. Geim, M. Lozada-Hidalgo, P. R. Unwin

**Affiliations:** 1 Department of Chemistry, University of Warwick, Coventry, UK; 2 Department of Physics and Astronomy, The University of Manchester, Manchester, UK; 3 National Graphene Institute, The University of Manchester, Manchester, UK; 4 Department of Photonics, Izmir Institute of Technology, Urla, Turkey; 5 Departement Fysica, Universiteit Antwerpen, Antwerp, Belgium; 6 Departamento de Fisica, Universidade Federal do Ceara, Fortaleza, Brazil

**Keywords:** Chemical physics, Graphene

## Abstract

Defect-free graphene is impermeable to all atoms^1–5^ and ions^6,7^ under ambient conditions. Experiments that can resolve gas flows of a few atoms per hour through micrometre-sized membranes found that monocrystalline graphene is completely impermeable to helium, the smallest atom^2,5^. Such membranes were also shown to be impermeable to all ions, including the smallest one, lithium^6,7^. By contrast, graphene was reported to be highly permeable to protons, nuclei of hydrogen atoms^8,9^. There is no consensus, however, either on the mechanism behind the unexpectedly high proton permeability^10–14^ or even on whether it requires defects in graphene’s crystal lattice^6,8,15–17^. Here, using high-resolution scanning electrochemical cell microscopy, we show that, although proton permeation through mechanically exfoliated monolayers of graphene and hexagonal boron nitride cannot be attributed to any structural defects, nanoscale non-flatness of two-dimensional membranes greatly facilitates proton transport. The spatial distribution of proton currents visualized by scanning electrochemical cell microscopy reveals marked inhomogeneities that are strongly correlated with nanoscale wrinkles and other features where strain is accumulated. Our results highlight nanoscale morphology as an important parameter enabling proton transport through two-dimensional crystals, mostly considered and modelled as flat, and indicate that strain and curvature can be used as additional degrees of freedom to control the proton permeability of two-dimensional materials.

## Abstract (web summary)

A study using high-resolution scanning electrochemical cell microscopy attributes proton permeation through defect-free graphene and hexagonal boron nitride to transport across areas of the structure that are under strain.

## Main

Measurements of proton transport through two-dimensional (2D) crystals demonstrated that these crystals pose an energy barrier for incoming protons of about 0.8 eV and about 0.3 eV for graphene and hexagonal boron nitride (hBN), respectively^8^. Additional experiments with hydrogen’s heavier isotope deuterium revealed that the initial energy of incoming protons is not given by thermal excitations (about 25 meV) but is instead about 0.2 eV owing to zero-point oscillations of protons bound to oxygen atoms in the proton-conductive media^9^. This correction lifts the total energy barriers, *E*, posed by the crystals to about 1.0 eV and about 0.5 eV for graphene and hBN, respectively. Despite these insights, the mechanism for proton permeation through the 2D crystals remains controversial. The general consensus from density functional theory calculations is that the energy barriers should be notably larger^14^. The studies (for example, refs. ^10,11,13,14,18^) have yielded a rather wide range of *E* but always exceeding the value of about 1 eV found experimentally. The spread of values arises from the various assumptions made in the models, such as whether the process is slower than the lattice relaxation timescale^14^, protons tunnel quantum mechanically^11,12^ or protons locally hydrogenate the carbon lattice (and hence locally expand it) before transfer^13,19^. This uncertainty has motivated an alternative explanation widely speculated in the literature, namely that proton permeation takes place through structural defects in the crystal lattice. This hypothesis is based on experiments using graphene grown by chemical vapour deposition (CVD)^15–17^, which has grain boundaries, pinholes and other imperfections that appear during growth and transfer^20–22^. Experiments using CVD graphene typically report very high proton permeation rates and, sometimes, even the loss of graphene’s impermeability to other ions^16^. However, the explanation that assumes atomic-scale defects as the only proton conductive sites is inapplicable to mechanically exfoliated graphene. Indeed, transmission and tunnelling electron microscopy have failed to observe any vacancies or other atomic-scale imperfections for scans over relatively large areas of such crystals. Even more decisively, gas permeation experiments that can easily detect a single-angstrom-scale defect permeable to gases within micrometre-sized membranes^1,2,4,5^ detected none in exfoliated graphene and hBN monolayers^6^. Further experimental evidence is necessary to understand proton transport through defect-free 2D crystals and resolve the existing controversy.

**Fig. 1:**
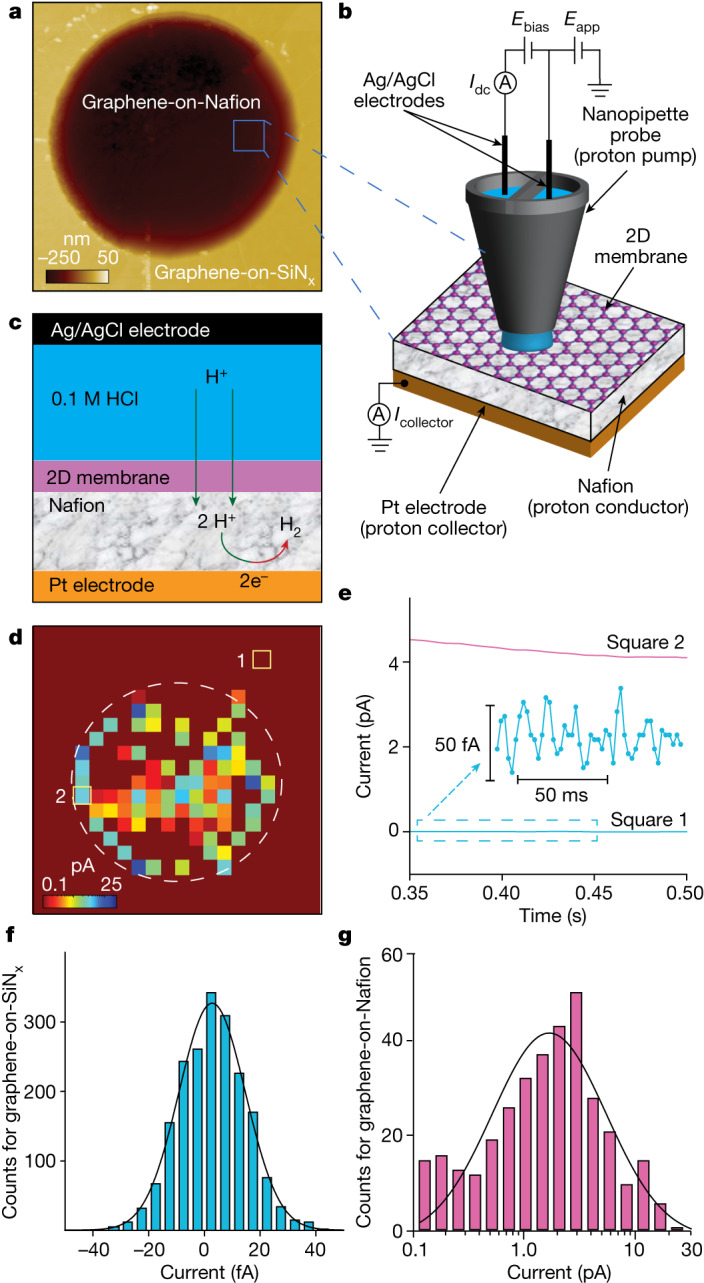
Nanoscale visualization of proton currents through 2D crystals. **a**, AFM image of one of our experimental devices, showing the 2-μm-diameter circular aperture in the SiN_x_ substrate covered with monolayer graphene. The bottom side of the membrane is in contact with Nafion (proton-conducting polymer) and the top side is left accessible to SECCM. **b**, Schematic of our SECCM setup. The blue square in **a** indicates the area zoomed-out into **b**. The nanopipette has two reservoirs filled with HCl electrolyte, each electrically connected using Ag/AgCl electrodes. Potential *E*_bias_ drives a current between these two electrodes (*I*_dc_). The current acts as a feedback signal that detects whether the probe is in contact with a sample surface. After a contact is established, the potential *E*_app_ is used to pump protons from the HCl reservoirs through graphene and onto the Pt electrode. This yields *I*_collector_, which is the current reported in the SECCM maps. The size of each measured pixel is given by the size of the nanopipette tip and the formed meniscus. Pixelated measurements are repeated over extended areas to generate a map (see the section in the Methods entitled SECCM scanning protocol). **c**, Schematic of proton flow through the devices. Protons are injected from the nanopipette through graphene into Nafion. After reaching the Pt proton collector, they evolve into H_2_ gas. **d**, Example of SECCM maps (*I*_collector_ maps) for apertures covered with graphene. The white dashed circle marks the aperture in SiN_x_. Colour scale bar, current in picoamperes. **e**, Steady-state SECCM currents as a function of time for graphene-on-Nafion (pink) and graphene-on-SiN_x_ (blue). The measurements are from the areas indicated by squares in **d** (colour coded). **f**, Statistical distribution of the measured values of steady-state proton currents for graphene-on-SiN_x_. The peak-to-peak noise level is about 50 fA (inset of **e**), which reduces to about 10 fA after time averaging. **g**, Similar statistics for graphene-on-Nafion. The SECCM currents were measured over the entire area shown in the maps, including the regions shown as brown pixels for both graphene-on-Nafion and graphene-on-SiN_x_ (see **d**). In **f**,**g**, each count represents the average of steady-state current over 100 ms. Data are collected from six different devices. Solid curves, best Gaussian fits.

**Extended Data Fig. 1:**
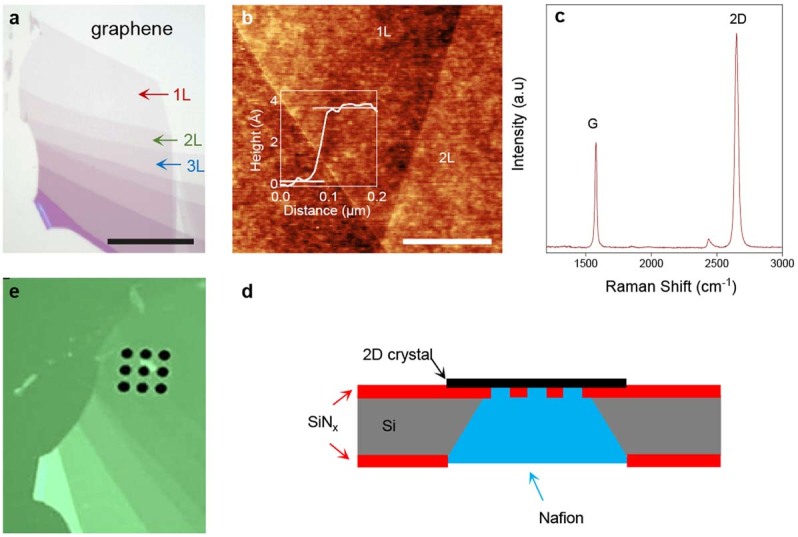
Experimental devices. **a**, Optical image of a typical flake obtained by mechanical exfoliation. The regions that are 1-, 2-, and 3- layers thick are marked with red, green and blue arrows, respectively. Scale bar, 20 μm. **b**, AFM image of a typical graphene flake placed on an oxidized Si wafer. The inset shows the step profile corresponding to one graphene layer. Scale bar, 1 μm. **c**, Raman spectrum for our typical graphene membrane. **d**, Schematic of our experimental devices. The 2D crystal is suspended over several apertures etched into a freestanding SiN_x_ film. The back side is coated with Nafion that is electrically connected to a Pt electrode. The top side is probed by SECCM. **e**, Optical micrograph of one our devices where the flake shown in panel **a** was transferred on its top as a membrane. The apertures (2 µm in diameter) are visible as black circles.

**Extended Data Fig. 2:**
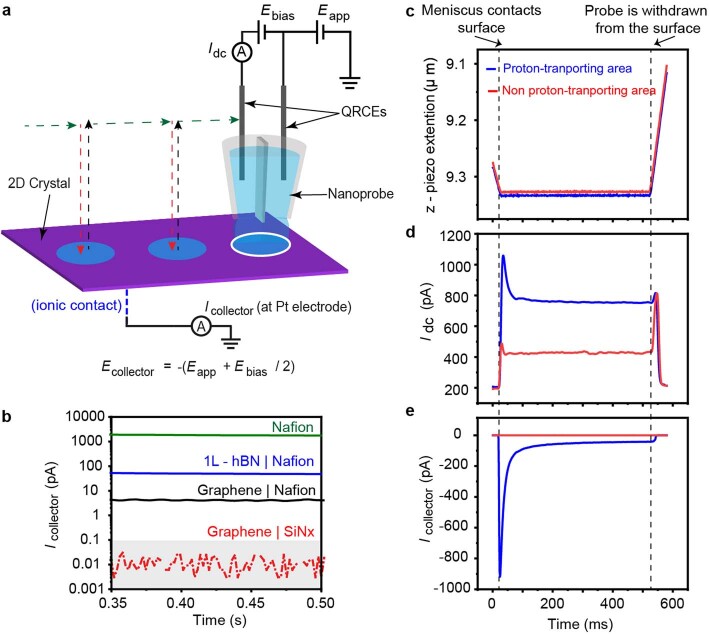
SECCM measurements. **a**, Their basic principle. A nanopipette with two reservoirs filled with 0.1 M HCl and fitted with identical quasi-reference electrodes (QRCEs) is used to inject protons through 2D crystals. A voltage applied between the QRCEs produces an ion current *I*_dc_ that is used as a feedback signal. Another potential (*E*_app_) applied to one of the QRCEs determines the overall potential used to inject protons. Dashed arrows illustrate the scanning protocol: the probe approaches the crystal (red) and makes contact over the area marked by blue circle, it is then retracted (black arrows) and moved to another position (green). **b**, Steady state SECCM currents collected from various settings: green, without any crystal covering Nafion; blue, monolayer hBN on top of Nafion; black, monolayer graphene on Nafion; and red, the same graphene monolayer on SiN_x_ away from apertures. This panel demonstrates that our experimental setup can probe proton conductivity of 2D materials within a 4-decade current range. **c**, Example of distance changes between the nanopipette and the sample during a typical scan obtained above proton-conducting (blue) and proton-blocking areas (red). **d**, *I*_dc_ - *t* characteristics measured simultaneously show that *I*_dc_ accurately detects the moments when meniscus wetting and dewetting occur. **e**, Corresponding *I*_collector_-*t* characteristics (same colour coding). Dotted lines in panels **c**–**e** mark the moments when the probe wets and retracts from the surface.

**Extended Data Fig. 3:**
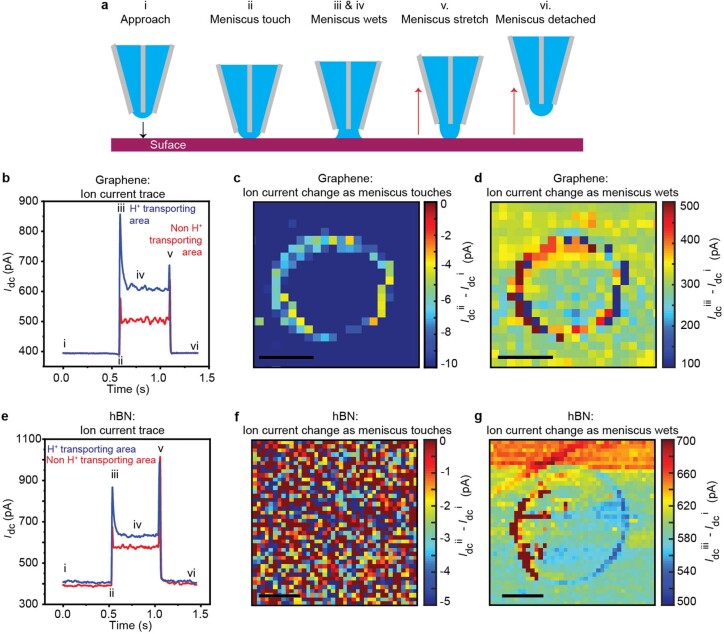
Meniscus-surface contact. **a**, Schematic of the approach and retract stages for our scanning protocol. **b**, Example of *I*_dc_ - *t* characteristics (ion current between the two electrodes in the nanopipette) for proton conducting (blue) and non-conducting (red) areas in a graphene device. **c**, Map showing changes in *I*_dc_ during step *ii* with respect to the current in step *i* (Δ*I*_dc_ = ${I}_{{\rm{dc}}}^{{\rm{ii}}}$ − ${I}_{{\rm{dc}}}^{{\rm{i}}}$) for the graphene device in **b**. **d**, Map of Δ*I*_dc_ = ${I}_{{\rm{dc}}}^{{\rm{iii}}}$ − ${I}_{{\rm{dc}}}^{{\rm{i}}}$ for the same device. **e**–**g**, Same as in panels **b**–**d** but for a monolayer hBN device. Scale bars, 1 μm.

In this report, we investigate the distribution of proton currents through mechanically exfoliated 2D crystals with high spatial (nanoscale) and high current (fA) resolution using scanning electrochemical cell microscopy (SECCM). The devices for this study consisted of graphene and hBN monolayer crystals, which were suspended over micrometre-sized holes (2 μm in diameter) etched into silicon nitride (SiN_x_) substrates (Methods and Extended Data Fig. 1). No structural defects are expected in the 2D crystals, as dozens of similar membranes were studied in ultrasensitive gas flow experiments, with none showing any permeation of helium^2^ (see the section in the Methods entitled Absence of defects in mechanically exfoliated 2D membranes). One side of the obtained free-standing 2D membranes was coated with a proton-conducting polymer (Nafion) that was in turn electrically connected to a millimetre-sized Pt electrode. The opposite side of the 2D crystal was left exposed to air and probed using SECCM (Extended Data Fig. 2). For SECCM measurements, a nanopipette with a tip opening diameter of about 200 nm and filled with 0.1 M HCl was accurately positioned over the sample using piezo drivers (Fig. 1a,b). On contact with the surface, a droplet meniscus was formed, whose size determines the surface area being probed (Extended Data Figs. 2 and 3). During such measurements, protons from the HCl reservoir in the pipette are injected through the sample with the potentials *E*_app_ and *E*_bias_ set so as to fix *E*_collector_ = −0.5 V at the Pt electrode (H^+^ collector) with respect to Ag/AgCl (see the section in the Methods entitled Scanning protocol). Therefore, the 2D crystal constitutes an atomically thin barrier between the SECCM probe (H^+^ pump) and the Nafion–Pt collector, and a current (*I*_collector_) is detected only when the probe is at locations where H^+^ transmission occurs (Fig. 1). This barrier is the current-limiting element in our devices, as corroborated directly by measuring the SECCM response at areas without 2D crystals (bare Nafion), which give currents >3 orders of magnitude higher (Extended Data Fig. 2).

**Extended Data Fig. 4:**
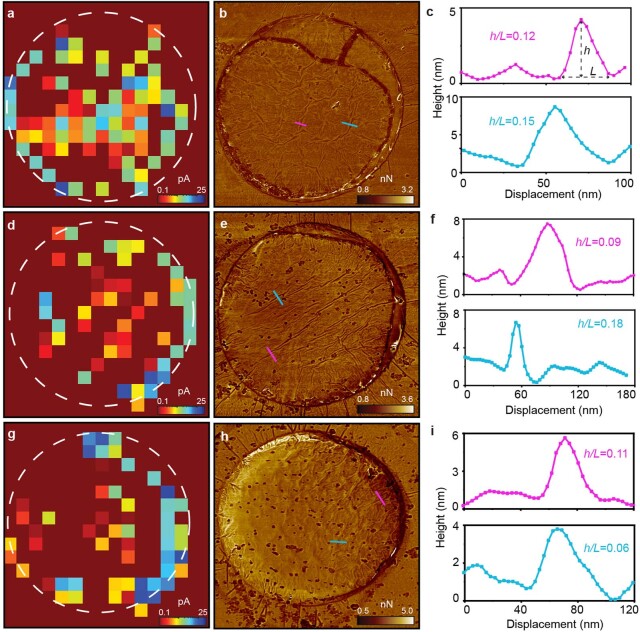
Additional examples of our proton-transport measurements. **a**,**d**,**g**, SECCM maps for 3 graphene devices. White dashed circles mark the 2-μm-diameter apertures in SiN_x_. **b**,**e**,**h**, AFM adhesion maps for the devices shown on the left panels. **c**,**f**,**i**, Height profiles for some wrinkles marked on the corresponding AFM maps (colour coded).

In the SECCM measurements, we acquire current versus time curves for each spatial location tested. These curves exhibit resistor–capacitor decay characteristics and a steady state is achieved typically within about 400 ms after meniscus formation at the sample surface (Fig. 1 and Extended Data Fig. 2). All of the SECCM maps presented below are in the steady state. Figure 1d shows an example of such maps obtained from monolayer graphene. If the device is scanned over areas of graphene covering the SiN_x_ substrate, only small parasitic (leakage) currents of about 10 fA are observed because the SiN_x_ substrate blocks proton transport (Fig. 1e,f). By contrast, for the areas where graphene is in direct contact with Nafion, proton currents of up to several picoamperes are observed. Notably, the SECCM maps (Fig. 1d and Extended Data Fig. 4) show that proton transport through graphene is spatially highly inhomogeneous, and this was the case for all of the studied devices (more than 20). Whereas several pixels inside graphene-on-Nafion areas show currents within our background noise, statistics for the other pixels exhibit a log-normal distribution with its mode located at about 2 pA, two orders of magnitude above the noise level (Fig. 1g).

**Extended Data Fig. 5:**
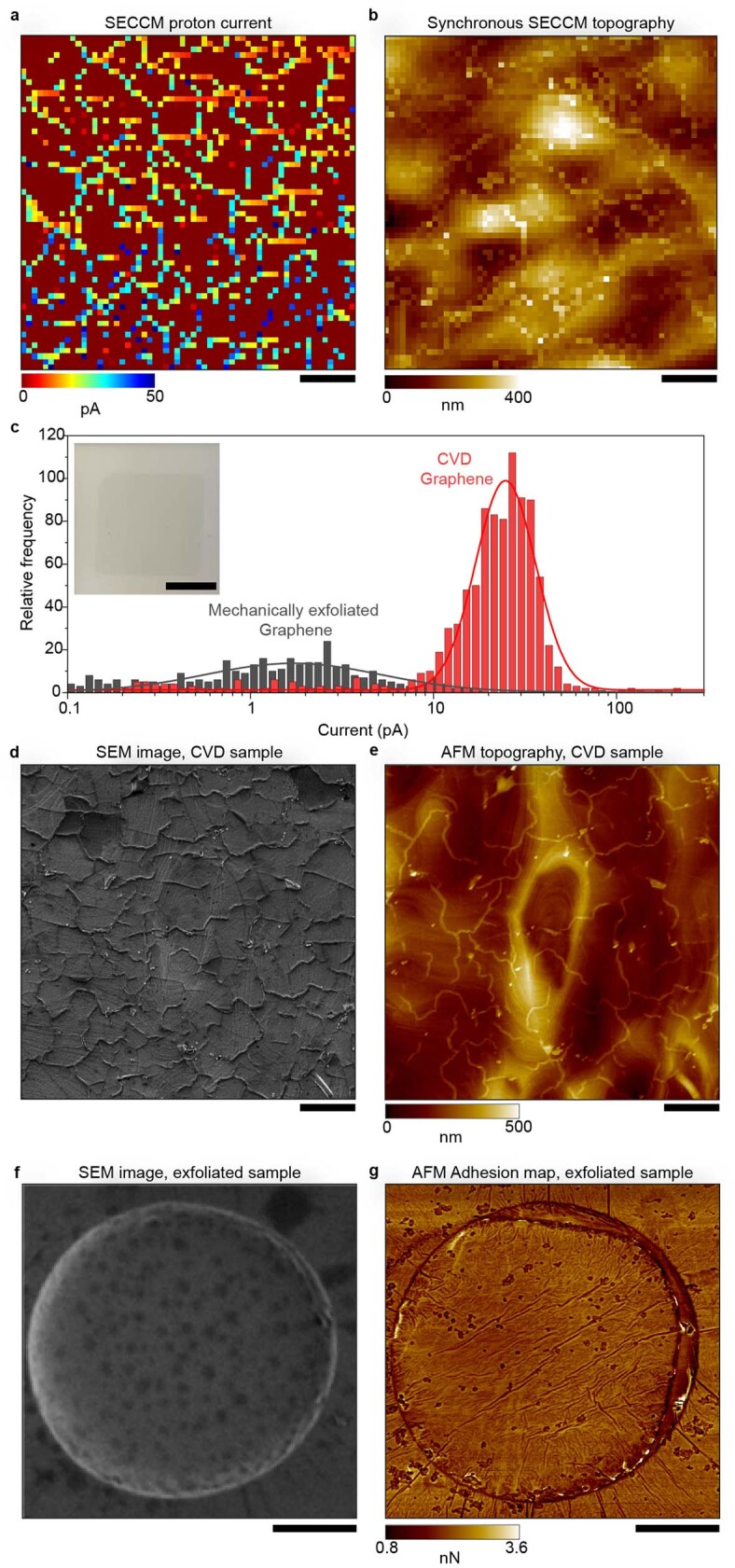
Proton transport through CVD graphene. **a**, SECCM map for a typical region within our CVD device. The map shows proton-permeation through a graphene area of ∼120 × 120 μm^2^. No single pixel with current above 100 pA was found. **b**, Topography of the area collected simultaneously with the SECCM scan in panel **a**. **c**, Statistics for steady-state proton currents from panel **a** (red bars) and for mechanically exfoliated graphene (grey bars). The CVD graphene data show a log-normal distribution with the peak at ∼20 pA and a long tail that matches typical proton currents observed for mechanically exfoliated graphene. Solid curves, best Gaussian fits to the data. Inset, optical image of CVD graphene on Nafion. Graphene is visible as a slightly darker square. Scale bar, 1 cm. **d**, Scanning electron micrograph of a typical area of this CVD graphene sample. Grain boundaries in the polycrystalline film are clearly visible. **e**, AFM image of the same area as shown in panel **d**. The scale bars in panels **a**,**b**,**d** and **e** are 20 μm. **f**, Scanning electron micrograph of a typical mechanically-exfoliated graphene membrane coated with Nafion from the back side. Areas with different (brighter and darker) contrast arise because of the polymer’s porosity, which is evident under higher magnification, as previously reported^50^. **g**, AFM adhesion map for the device in panel **f**. Scale bars in panels **f** and **g**, 1 μm.

It is instructive to compare these results with measurements on similar devices but made from CVD graphene. Previously^15–17^, proton transport through CVD graphene was attributed to sparsely distributed defects (probably microholes; one per 10^3^–10^4^ μm^2^), each exhibiting a proton current^16,17^ of about 0.3–1 nA under similar *E*_bias_. Our present SECCM measurements using higher-quality CVD graphene have not found such isolated highly conductive defects (possibly pinholes) and instead show enhanced permeation over large areas, mostly at what seems to be grain boundaries, a common feature of CVD-grown graphene^7,20^ (Extended Data Fig. 5, and the section in the Methods entitled SECCM of CVD graphene membranes). The SECCM currents exhibit a log-normal distribution with the mode (peak) at about 20 pA. This is two orders of magnitude lower than the current through individual defects found previously in CVD graphene but an order of magnitude higher than that in our mechanically exfoliated graphene monocrystals. In this context, exfoliated graphene is fundamentally different from CVD graphene. Indeed, neither nanoscale holes nor grain boundaries are present in our monocrystals but the experimental resolution still allows us to observe about 100 proton-conductive sites per square micrometre. As a result of the proven gas and ion impermeability of mechanically exfoliated graphene^2,5^, these sites cannot be attributed to atomic-scale defects, not even vacancies^5^. Accordingly, we must conclude that a defect-free graphene lattice is proton permeable, in agreement with earlier conclusions^8,9^. In the rest of this report, we explore the origins of the unexpected spatial inhomogeneity of proton transport through defect-free monolayers of graphene and hBN.

**Fig. 2:**
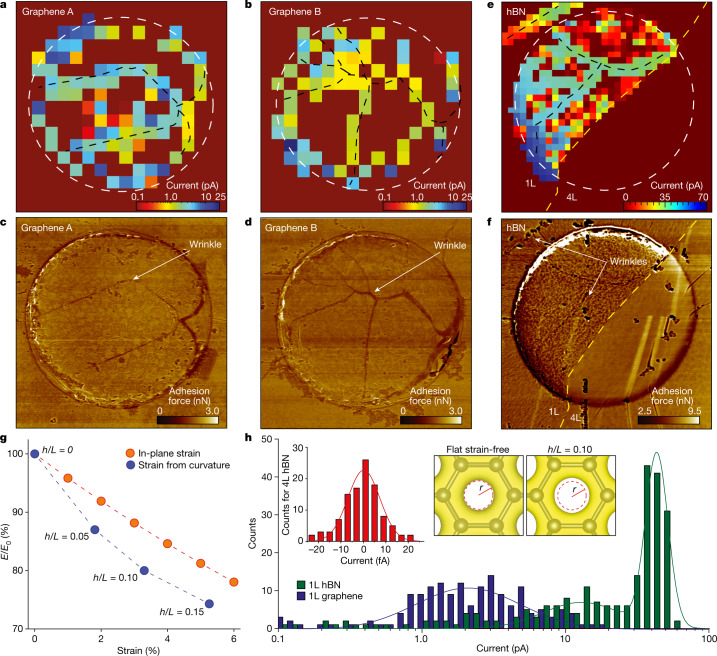
Unexpected inhomogeneity of proton transport through 2D crystals. **a**,**b**, SECCM maps for two graphene devices. The white dashed circles mark the rim of the 2-μm-diameter apertures in SiN_x_. **c**,**d**, AFM force maps for the devices in the panels above. Wrinkles and edges are clearly visible in the AFM maps and correlate with high-conductivity areas in the SECCM maps. For easier comparison, the black dashed curves in **a** and **b** mark wrinkles’ positions. **e**, Proton currents through an hBN device. Yellow dashed curve, border between monolayer (1L; left) and tetralayer (4L; right) hBN. **f**, AFM force map for the device in **e**. Apparent wrinkles are indicated by the arrows and marked by the black dashed curves in **e**. A particular feature of this device is notable proton currents in the top left corner in **e**, away from the aperture in SiN_x_. Extended Data Fig. 6 reveals that this feature is due to a wrinkle originating from a neighbouring aperture. The wrinkle provides a nanocavity between hBN and the SiN_x_ substrate, which allows protons to reach this area. **g**, Strain lowers the energy barrier *E* for proton permeation (*E*_0_ is the barrier for unstrained graphene). Blue symbols, the effect of strain arising from curvature; values of *h*/*L* are specified next to each point. Red data, *E*/*E*_0_ due to purely in-plane strain. **h**, Statistics of proton currents for graphene and hBN monolayers (data from **a**,**b**,**e**). Left inset, statistics collected from the tetralayer region. Solid curves, best Gaussian and double-Gaussian fits for graphene and monolayer hBN, respectively (accuracy of about 10% in determining the modes of the normal distributions). The right two-panel inset shows the calculated electron density provided by the crystal lattice for unstrained (left) and strained (right) graphene; the latter calculations are for strain arising from curvature with *h*/*L* = 0.10. To make changes in the electron density evident, the dashed red circle in the left panel marks the boundary between regions^8^ with densities above and below 0.2 *e* Å^−3^ (the latter region is shown in white). The same circle is projected onto the right panel and emphasizes that the low-density region expanded in the strained lattice.

To understand the observed spatial inhomogeneity of SECCM maps, we compare them with atomic force microscopy (AFM) images of the 2D crystals. Figure 2a–d shows AFM adhesion force maps and corresponding SECCM scans for two graphene devices (for more examples, see Extended Data Fig. 4). The AFM micrographs reveal that the membranes are not flat but contain wrinkles that are a couple of nanometres in height, *h*, and tens of nanometres in width, *L* (*h*/*L* ≈ 0.06–0.18, as found from topography maps; Extended Data Fig. 4). It is clear from Fig. 2a–d that the positions of the wrinkles closely correlate with some of the most highly conductive regions in the SECCM maps (blue pixels). Other areas of high proton conductivity occur around the apertures’ rims. The common denominator for the two types of high-conductivity region is that, in both cases, the 2D membrane is under notable strain. Although the whole membrane is strained from being suspended, the stress mainly accumulates around the rim^23^. The resultant tensile strain is estimated at a few percent (see the section in the Methods entitled AFM and scanning electron microscopy characterization). Stress is also known to accumulate near wrinkles^24^, which can result in strain similar to that around the aperture’s rim.

**Extended Data Fig. 6:**
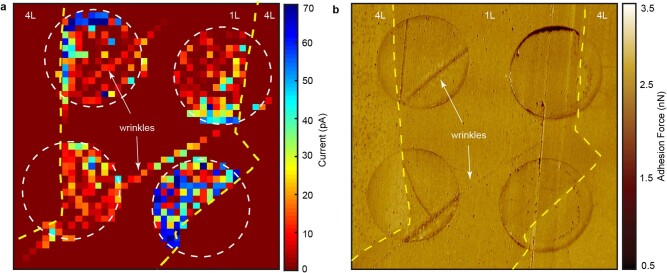
Additional SECCM maps for hBN. **a**, Four apertures covered with mono- and tetra- layer hBN. Dashed white circles mark the apertures. Their diameter, 2 µm. The yellow dashed lines show the border between areas covered with 1L and 4L hBN. The device in the lower right corner is the one discussed in Fig. 2 of the main text. **b**, AFM force maps for the devices in **a**. Two wrinkles that facilitate proton transport above the silicon-nitride substrate (away from the apertures) can be seen to originate from the two left devices.

Next, we describe similar experiments with devices fabricated using monolayer hBN instead of graphene. Figure 2e shows one of our hBN devices, in which half a SiN_x_ aperture is covered with monolayer hBN and the other half with tetralayer (additional examples in Extended Data Fig. 6). The areas covered with the tetralayer are notably flatter than those with monolayer and exhibit no proton transport within our resolution, even at high-strain areas around the rim. This is in agreement with our previous work in which no proton permeability could be detected for ≥4 layers of hBN^8^. By stark contrast, areas covered with monolayer hBN exhibit a high density of highly conductive sites with currents generally larger than in graphene devices. This is consistent with the fact that hBN monolayers are on average about 50 times more proton conductive than graphene^8,9^. As in graphene devices, the highest activity (blue pixels) in the SECCM map is concentrated around wrinkles and the rim. However, the maps for hBN monolayers also reveal many active areas that do not correspond to any obvious morphological features. In the statistical distribution (Fig. 2h), the corresponding currents result in a notable shoulder centred at about 10 pA, whereas currents from wrinkled areas are centred at about 50 pA. This allows us to estimate that wrinkles accelerate proton transport by a factor of about five with respect to that from featureless regions. Although no such shoulder was apparent for graphene membranes, some SECCM regions without morphological features also exhibited many active pixels with currents about 0.1–1 pA (well above the noise level). This may suggest a similar relation between current amplitudes for strained and featureless regions in graphene membranes.

We attribute the smaller proton currents away from apparent morphological features to transport through nanoscale ripples that are ubiquitous in 2D crystals^2,25–27^. These ripples can be either dynamic (flexural phonons in freely suspended membranes) or static (caused by strain or adsorbates), as previously revealed by transmission electron microscopy^26^. Although our AFM maps do show a difference in apparent roughness for monolayer and tetralayer regions in Fig. 2f that can be attributed to static ripples, these ripples are too small (*L* of several nanometres^25,26^) to be quantified using our AFM. Nevertheless, direct evidence from transmission electron microscopy has previously found^25,26^ that static ripples have typical *h*/*L* ≈ 0.1, comparable to the aspect ratios observed for the wrinkles. On this basis, we propose that unavoidable nanorippling of 2D crystals enhances their proton permeability in much the same way as larger wrinkles. As nanoripples have smaller *L*, their smaller areas result in smaller proton currents within individual pixels. To corroborate this microscopic picture provided by SECCM, we integrated the observed currents over the entire area of the 2D membranes. This allows for an estimate of the proton conductivities of graphene and hBN monolayers as about 10 mS cm^−2^ and about 300 mS cm^−2^, respectively. Taking into account our experimental uncertainty (factor of about three that comes mostly from assessing the area contacted by the probe during the scans), the found conductivities are in good agreement with the previous measurements of average proton conductivities of the 2D crystals^8^.

**Extended Data Fig. 7:**
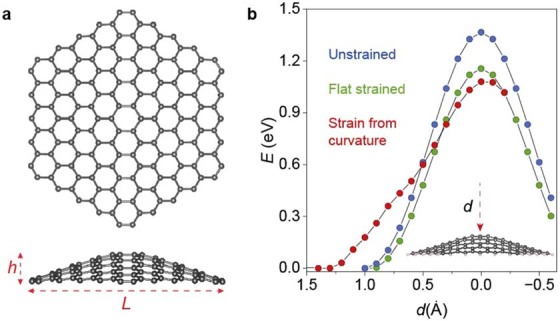
DFT calculations. **a**, Simulated structure of rippled graphene. Its plan and side views (top and bottom, respectively). **b**, Energy barrier for proton penetration for strain-free graphene (blue), flat graphene with biaxial strain of 3.3% (green) and rippled graphene with strain arising from curvature (*h*/*L* = 0.10, *ε* = 3.3%). Calculations for *E* were done using 0.1 Å steps for the distance *d* from graphene as shown in the inset.

Our density functional theory calculations (Methods) provide further support for the above explanation of localized strain as the main reason for the observed spatial inhomogeneity in proton transport. Indeed, the barriers imposed by the 2D crystals for incoming protons depend on the density of electron clouds associated with the crystal lattice^8^. For example, monolayer hBN presents a sparser electron density than graphene and, accordingly, is more permeable to protons^8^. Strain and curvature modify the electron-density distribution within 2D materials (Fig. 2h, right two-panel inset), and this can enhance their transparency to protons. Our calculations show that, by stretching the graphene lattice by about 5%, the energy barrier *E* for protons is lowered by about 20% (Fig. 2g and Extended Data Fig. 7). If this strain is accompanied by curvature (like in the case of ripples), the barrier is reduced further, so that *E* reaches about 75% of the unstrained value *E*_0_ (Fig. 2g). Although this reduction seems relatively small, proton currents depend exponentially on the barrier height^8^, which means that proton transport can be accelerated by several orders of magnitude within strained regions around wrinkles, ripples and other morphological features.

In conclusion, our experiments show that strain-inducing morphological features in otherwise defect-free 2D crystals are associated with enhanced proton conductivity around them. A notable example of this is graphene wrinkles that do not require any crystal-lattice defects but result in high proton currents, not dissimilar to the case of grain boundaries in CVD graphene (Methods). Our findings also suggest that nanoscale ripples, ubiquitous in 2D membranes and known to result in considerable strain, accelerate proton transport within nominally flat areas. This is important as graphene is typically modelled as a perfectly flat unstrained crystal. As strain and curvature in 2D membranes can typically reach up to 10%, the theories predicting *E*_0_ of up to 1.5 eV for flat unstrained graphene (for example, refs. ^10,14^) seem to be consistent with the experiments reporting barriers of about 1.0 eV (ref. ^8^). Finally, strain and curvature can be exploited to enhance proton conductivity of 2D crystals, which is of interest for various applications involving proton transport^9,28–30^.

## Methods

### Device fabrication

Micrometre-sized apertures were etched into silicon-nitride-coated silicon substrates (500 nm SiN_x_) using photolithography, wet etching and reactive ion etching, following the protocol previously reported^8^. Our devices had several apertures next to each other and were 2 μm in diameter each (Extended Data Fig. 1). Monolayers of graphene and hBN were obtained by micromechanical cleavage^31^ and identified using a combination of optical microscopy, AFM and Raman spectroscopy, as previously reported^8,32,33^. The monolayers were suspended over the apertures in the SiN_x_ substrate. The resulting free-standing membranes were coated on one side by drop-casting the Nafion polymer (5%, 1,100 equivalent weight) to obtain ≈10-μm-thick films. The devices were annealed in a water-saturated environment at 130 °C to crosslink the polymer. The collector electrode was prepared by laminating a Pt foil (10 × 10 mm, 99.95% purity, Goodfellow) onto a cylindrical carbon block with a hot compression mounting machine (SimpliMet). The exposed Pt surface was then subjected to mechanical and electrochemical polishing. Gaskets were used to cover the carbon block and expose only the Pt surface for contact with Nafion–2D crystal devices^34^. For measurements, the Nafion film was hydrated with deionized water and allowed to equilibrate before placing it in contact with the Pt collector.

### SECCM probes

Nanopipettes for SECCM were fabricated from quartz theta capillaries with filaments (WAR-QTF120-90-100, Friedrich & Dimmock). The capillary (outer diameter, 1.2 mm; inner diameter, 0.90 mm; length, 100 mm) was pulled to form a fine sharp point with a tip opening diameter of about 200 nm, using a CO_2_-laser puller (Sutter Instruments P-2000). The nanopipette was then filled with 100 mM HCl electrolyte, and a silicone oil layer was added on top of the electrolyte solution in the tip to minimize evaporation during prolonged scanning procedures^35^. Two AgCl-coated Ag wires, fabricated by electrochemically oxidizing Ag wires (0.125 mm in diameter) in saturated KCl solution^36^, were used as quasi-reference counter electrodes (QRCEs). Each of the nanopipette channels was fitted with a QRCE positioned about 3–4 cm away from the tip end^36^.

### SECCM instrumentation

SECCM was carried out using a home-built workstation^37^. The SECCM probe was mounted on a *z* piezoelectric positioner (P-753.1CD LISA, Physik Instrumente) whereas the studied graphene or hBN device was mounted on an *x*–*y* piezoelectric positioner (P-622.2CD PIHera, Physik Instrumente). The SECCM probe was moved to the initial scan position using an *X*–*Y*-micropositioner (M-461- XYZ-M, Newport) controlled with Picomotor Actuators (8303, Newport). An optical camera provided a visual guide for the probe positioning. The microscopy stage and all positioners were enclosed in a Faraday cage with heat sinks and vacuum panels to minimize noise and thermal drift. The Faraday cage was placed on an optical tabletop with tuned damping (RS 2000, Newport) balanced on a high-performance laminar flow isolator (S-2000 Series, Newport).

Data acquisition and instrumental control were carried out using an FPGA card (PCIe-7852R) running the Warwick Electrochemical Scanning Probe Microscopy software (https://www.warwick.ac.uk/electrochemistry/wec-spm). Two home-built electrometers were used for current measurements, together with home-built eighth-order brick-wall filters with the time constant of the current amplifier set to 10 ms. Influence of acquisition parameters on the noise level is detailed elsewhere^38^. The current data were acquired every 4 μs, and 256 samples were averaged to give a data acquisition rate of about 1 ms.

### Scanning protocol

SECCM was deployed in the hopping mode^34,39,40^ by recording spatially resolved current versus time (*I*–*t*) traces of the reduction of protons at the bottom Pt collector electrode (Extended Data Fig. 2). The hopping mode protocol involved the approach of the probe to the 2D crystal surface until the electrolyte meniscus at the end of the tip (not the nanopipette itself) made contact (as detailed below). After a measurement on one site was completed, the probe was retracted and moved to the next site to generate a map of *I*–*t* traces over the entire device surface.

Two voltage controls are important in this protocol. The first is the potential difference, *E*_bias_, between the QRCEs in each of the two channels of the nanopipette (Extended Data Fig. 2a). This gives rise to an ion current between the two channels in the nanopipette, *I*_dc_, which is used as a feedback signal to detect whether the meniscus is in contact with the surface. When the droplet meniscus touched the device surface, a spike in *I*_dc_ ≫ 100 pA signified ‘jump-to-contact’^41^ (detected with a feedback threshold of 45 pA; Extended Data Fig. 2b,c). When this threshold was reached, the probe motion was stopped. Note that this signal provided a means of landing the meniscus on the 2D crystal surface, irrespective of its local proton permeability. Further details of the *I*_dc_ transients are provided in the section entitled Consistency of meniscus-surface wetting.

The second voltage, *E*_app_, between the nanopipette probe and the proton-collecting working electrode sets the potential of the Pt collector electrode with respect to the QRCEs as *E*_collector_ = −(*E*_app_ + *E*_bias_/2) (ref. ^42^). This potential is chosen as *E*_collector_ = −0.5 V versus Ag/AgCl QRCE (equivalent to an overpotential of about 0.2 V versus the standard potential for the hydrogen evolution reaction). This was the maximum voltage used in ref. ^8^ in which the linear-response proton transport was studied in similar devices. *E*_collector_ drives the electrochemical proton reduction at the Pt electrode, which results in current *I*_collector_ (inset of Extended Data Fig. 2). *I*_collector_–*t* measurements were made for 500 ms and involved the area defined by the meniscus between the SECCM nanopipette and the substrate. After each measurement within this temporary and spatially localized droplet cell, the probe was retracted at a speed of 4 µm s^−1^ (Extended Data Fig. 2b) and moved to the next location where the above procedure was repeated. This allowed us to obtain a spatial- and time-resolved dataset for *I*_collector_. Proton-current maps in our figures are presented as an average of the last 100 ms of the *I*_collector_–*t* transients.

The *z*-position of the probe was recorded synchronously throughout the whole measurement procedure, with the value at the end of each nanopipette approach yielding a topographical map of the studied 2D-crystal device. Nonlinear sample-tilt and piezo-drift effects in such topographical maps were corrected using the scanning probe image processing software package (v6.0.14, Image Metrology). SECCM topographical maps of mechanically exfoliated graphene devices (not shown) were similar to those subsequently obtained by AFM, as expected. Such maps, recorded synchronously with proton transport activity, were especially valuable as they revealed morphology around proton-conducting sites in CVD graphene (Extended Data Fig. 5).

### Consistency of meniscus-surface wetting

The SECCM maps of proton transport through graphene and hBN monolayers exhibit a marked spatial inhomogeneity. To establish that this is an intrinsic property of the 2D crystals and not a result of variations in surface–probe contact, we investigated the consistency of meniscus-surface wetting by analysing the current *I*_dc_ flowing between the two channels in the nanopipette. Below we explain how *I*_dc_ is used as a feedback signal that unequivocally detects meniscus-surface wetting, regardless of proton transport through a 2D crystal.

Extended Data Fig. 3 illustrates the steps that take place during the SECCM scan and how *I*_dc_ changes during each step. Initially, the probe is not in contact with the sample (step i (approach)) and ${I}_{{\rm{dc}}}^{{\rm{i}}}$ is constant as a function of time (about 400 pA in this case). As the probe gets closer, the meniscus encounters the sample (step ii, (meniscus touch)). In this step, ${I}_{{\rm{dc}}}^{{\rm{ii}}}$ sometimes decreased very slightly with respect to ${I}_{{\rm{dc}}}^{{\rm{i}}}$ (Δ*I*_dc_ = ${I}_{{\rm{dc}}}^{{\rm{ii}}}$ − ${I}_{{\rm{dc}}}^{{\rm{i}}}$ < 0), attributed to slight squeezing of the meniscus. However, this depended on a specific 2D material measured. For graphene samples, we typically see a decrease of about 1% or 5 pA (Extended Data Fig. 3b,c), whereas for hBN we do not see such a drop (Extended Data Fig. 3e,f). We attribute this difference in behaviour to a stronger attraction of the electrolyte in the probe to hBN than graphene. The next step is meniscus wetting (step iii (meniscus wets)). This step is characterized by a sharp increase in current, Δ*I*_dc_ = ${I}_{{\rm{dc}}}^{{\rm{iii}}}$ − ${I}_{{\rm{dc}}}^{{\rm{i}}}$ > 200 pA, which is an unmistakable indicator that the meniscus has fully wetted the sample (Extended Data Fig. 3b,d and Extended Data Fig. 3e,g for graphene and hBN, respectively). The d.c. current then drops to a steady state (step iv) during which the meniscus stabilizes. After the pre-programmed measurement period (500 ms of meniscus contact), the tip is retracted (step v (meniscus stretch) and step vi (meniscus detached)), with *I*_dc_ first sharply increasing and then returning to the initial value. These steps were clearly visible throughout scanning of entire samples.

The described behaviour was observed independently of *I*_collector_ (that is, whether the proton current is being pumped or not through the device into the proton collector). Extended Data Fig. 3b,c also show that *I*_dc_ exhibits the same features both in areas of high proton conductivity (blue curve) and in areas where no proton transport takes place (red). This shows that meniscus wetting of the sample is independent of proton transport through 2D crystals. Note, however, that the magnitude of *I*_dc_ does change for active and inactive areas because the Ag/AgCl electrodes are also the counter electrodes for proton conductivity measurements^42^. This change served as independent confirmation of those sites where there was notable proton permeation through 2D crystals.

Note that the above also rules out changes in the droplet cell size as the source of the observed spatial inhomogeneity of the proton currents. The consistency of the SECCM cell size across the surface is also in accord with the following considerations. First, wrinkles protrude at most a few nanometres from flat areas of graphene, which leads to only small variations in the involved surface area as compared to the area probed in each pixel (about 200 nm in diameter). Therefore, this cannot explain the orders of magnitude difference in the observed SECCM activity. Second, the roughness associated with the wrinkles is much less than a typical surface roughness of a wide range of samples previously studied by SECCM for which consistent meniscus cell size was observed or deduced^34,35,37,40,42–44^.

### AFM and scanning electron microscopy characterization

High-resolution topography and adhesion AFM imaging was carried out under ambient conditions with a Bruker Dimension Icon AFM using the PeakForce mode. The instrument was equipped with SCANASYST-AIR silicon tips (Bruker). The tips had a nominal spring constant, *k* = 0.4 N m^−1^, resonant frequency of 70 kHz and a tip radius of 2 nm. The resulting AFM maps were used to estimate strain across different areas of the 2D membranes. From AFM traces through the membrane centre, we estimate that the membranes were globally strained by typically 0.5%. However, the strain *ε* was distributed not uniformly but accumulated around the aperture rim^23^, leading to *ε* several times higher than away from it^23^. This yields *ε* of a few percent around the rim. Such strain is also expected to accumulate around wrinkles in the 2D membranes, whose complex morphology cannot be attained using strain-free (bending alone) deformations^24,26^. From the height (*h*) and base (*L*) of the wrinkles measured in AFM, we also estimate strain of a few percent, consistent with the above expectations.

For scanning electron microscopy (SEM) characterization, we used a Zeiss Gemini500 scanning electron microscope, using an In-lens secondary electron detector, accelerating voltages of 0.5–2 keV and a working distance of about 2 mm.

### Additional examples of devices studied by SECCM

Extended Data Fig. 4 (graphene) and 6 (hBN) show further examples of SECCM and AFM maps. In all of the measured devices (more than twenty 2D membranes), we observed a clear correlation between high-activity areas in the SECCM maps and morphology of 2D membranes. In particular, Extended Data Fig. 6 provides an example in which proton conductivity becomes sharply suppressed crossing the boundary from monolayer hBN to a 4-layer region. Previously, it was shown that hBN monolayers were highly proton permeable, whereas hBN crystals of four or more layers in thickness exhibited indiscernible proton conductance^8^. The images of Extended Data Fig. 6 illustrate this property with nanoscale resolution across individual membranes in the same experiment. An additional notable feature seen in the AFM maps is two wrinkles that extend along the SiN_x_ substrate beyond individual apertures. These wrinkles exhibit notable proton-conducting activity in the SECCM maps that occurs not only above the Nafion region but extends onto the SiN_x_ substrate. We attribute this observation to water that fills the space between the substrate and wrinkles and thus provides a proton-conducting medium inside the wrinkles.

### Absence of defects in mechanically exfoliated 2D membranes

Suspended membranes made from exfoliated 2D crystals have previously been characterized extensively using AFM, SEM, Raman spectroscopy, transmission electron microscopy and scanning tunnelling microscopy^2,5,6,8,9,25,45,46^ as well as gas permeation measurements^1,2,4,5^. None of those studies could detect any structural defects in the membranes. Nevertheless, it was important to ensure that the fabrication procedures used in the present report did not lead to accidental tears, cracks or pinholes that would break the continuity of the graphene lattice and leak protons through.

The formation of wrinkles in supported thin sheets is a universal phenomenon that arises from non-uniform adhesion between the sheet and the substrate. For example, this phenomenon has been extensively studied for 2D polymers^47^, and graphene is no exception. To understand the formation of wrinkles in our devices we note that graphene sheets are initially suspended over holes, rather than supported. The membranes are therefore stretched laterally because of adhesion to the holes’ sidewalls and free to relax in the out-of-plane direction. In most cases, this results in wrinkle-free membranes^2^. The situation changes after depositing Nafion. Adhesion to sidewalls disappears in the presence of water (as observed in ref. ^48^) so that graphene is no longer stretched over the holes. The membrane therefore becomes looser, which unavoidably results in the formation of wrinkles. In addition, the now loose graphene sheets conform to the porous Nafion polymer surface, which further contributes to the wrinkling and rippling. Importantly, the wrinkles and roughness do not lead to cracks, tears or pinholes that would allow unimpeded proton permeation through them. This conclusion is supported by many experimental observations. For the sake of brevity, we describe below only three of them.

First is the Raman spectra observed for the wrinkled membranes on Nafion. Any defects in graphene leading to breakdown of its continuous crystal lattice (cracks, tears, holes or even individual vacancies) activate the so-called D peak in its Raman spectrum. The intensity of this peak increases with defect density (for example, refs. ^33,49^). Our graphene monocrystals do not exhibit any discernible D peak, which allows us to put an upper bound on the atomic-scale defect density of about 10^9^ cm^−2^. This translates into no more than 10 single-atom vacancies for our entire membranes of 2 μm in diameter (for example, refs. ^8,9,45^). By contrast, the reported wrinkles are hundreds of nanometres long, and if there were any breakdown of crystallinity along them, an intense D peak would also be apparent. Occasionally, we found devices with accidental cracks formed during fabrication, and those exhibited a strong D peak. They were discarded. All of the devices reported in the manuscript had no discernible D peak (Extended Data Fig. 1c). Also note that the found upper bound of about 10 atomic-scale defects in our devices cannot possibly explain the observed proton conductance. Indeed, our SECCM maps typically reveal about 100 active pixels and, to provide their proton conductance, not individual vacancies but large multi-atom pinholes would be required. This would lead to a very intense D peak, easily observable experimentally.

The second piece of evidence that rules out lattice defects in our membranes comes from measurements using liquid electrolytes (refs. ^6,8^). These experiments have found similar proton conductivity as in devices measured using Nafion. Unfortunately, we cannot remove Nafion after measurements, but we could remove electrolytes. In the latter case, the membranes did not show any D peak or any damage under AFM or SEM, which demonstrates that the membranes were not damaged during proton conductivity measurements. As the conductivity using electrolytes is the same as in the case of Nafion, we can safely conclude that Nafion does not damage graphene membranes either.

Finally, gas impermeability of our graphene–Nafion devices also proves the absence of defects induced by deposition of Nafion. Unlike graphene, which is completely impermeable to helium, thin Nafion films (after graphene was removed) exhibited notable helium leakage. This was measured using a He-leak detector that allowed us to resolve flows as low as 10^8^ atoms s^−1^. Nafion-coated graphene devices with an accidental crack exhibit notable helium permeability, whereas undamaged devices remain leak free, despite the presence of wrinkles.

### SECCM of CVD graphene

For these measurements, centimetre-scale pieces of CVD graphene (grown on Cu) were transferred onto a Nafion N212 film as reported previously^50^. To this end, the Cu foil that was covered on both sides with graphene was first exposed from one side to oxygen plasma, which removed graphene from that side. The CVD graphene remaining on the other side was then hot-pressed against the Nafion film, and the Cu foil was etched away in an ammonium persulfate solution. The resulting graphene-on-Nafion stack was left in deionized water for days to remove etchant residues. For SECCM measurements, centimetre-sized graphene-on-Nafion samples were fixed^17^ to the Pt electrode (as described above) and characterized using the same procedures as for micrometre-sized 2D crystals.

Extended Data Fig. 5 shows that proton currents detected by SECCM for our CVD graphene were below 100 pA. There were no spots with very high currents similar to those observed for lower-quality CVD graphene devices^15,17^. Statistics of the currents collected over large areas (Extended Data Fig. 5c) can be separated into two groups. The first group of pixels exhibits currents of 0.1–10 pA; this is similar to those found in mechanically exfoliated graphene reported in the main text. The second group of pixels exhibits a normal distribution with the mode at about 20 pA, which is about 10 times higher than currents in the first group. AFM and SEM images revealed that the higher-activity areas resulting in the second group came mostly from grain boundaries (Extended Data Fig. 5b–e). The higher permeability for these pixels can be attributed to multiple crystal-lattice defects present in grain boundaries (for example, 8-atom rings that are highly proton conductive^7,20^ or even bigger defects). We have also found that grain boundaries in CVD graphene were often accompanied by local corrugations (Extended Data Fig. 5d,e) with *h* ≈ 60 nm and *L* ≈ 500 nm, which may also have contributed to their proton permeability (*h*/*L* ≈ 0.1).

The experiments described in this section provide important insights into the large variability in proton permeability reported in the literature for CVD graphene films^51^. Even in the absence of gross defects (for example, cracks and tears), which sometimes are prevalent in CVD graphene films^17^, nanoscale pinholes can result in isolated hotspots with proton currents^17^ up to 1 nA. For higher-quality CVD graphene without such defects, proton conductivity is likely to be dominated by grain boundaries. Even in the last case, considerable variability in proton permeability is expected because of different grain sizes, depending on growth conditions, so that graphene films with smaller grains and thus higher density of grain boundaries would exhibit higher proton conductivity, in agreement with the previous report^7^.

### Density functional theory calculations

We used the projector augmented-wave method^52^ implemented in the Vienna ab-initio Simulation Package^53^ to model pseudopotentials of protons, and C and H atoms. The exchange-correlation potential was taken into account by considering the generalized gradient approximation within the Perdew–Burke–Ernzerhof form^54^. The weak van der Waals forces between graphene and proton were also included by using the DFT-D2 method of ref. ^55^. For geometry optimizations, a kinetic energy cutoff of 500 eV was used for the plane-wave basis. The convergence criterion of the total force on each atom was reduced to 10^−5^ eV Å^−1^ and the convergence criterion for the energy was set at 10^−6^ eV. For calculating the proton barrier, we used the proton pseudopotential from the hydrogen atom and then removed an electron from the whole system.

The flat and rippled graphene were simulated as a relatively large circular-shaped crystal consisting of 150 carbon atoms (about 22 Å in size), which was sufficient to prevent proton–proton interactions between neighbouring supercells. The carbon cells were isolated with a vacuum gap larger than 10 Å, which ensured the absence of edge-to-edge interactions. The ripples were modelled by fixing the out-of-plane positions of carbon atoms so that the crystal forms a Gaussian profile of height *h* (Extended Data Fig. 7). The atoms were allowed to relax in-plane. Interatomic distances for atoms near the ripple top (${d}_{cc}^{{\rm{s}}{\rm{t}}{\rm{r}}{\rm{a}}{\rm{i}}{\rm{n}}{\rm{e}}{\rm{d}}}$) were compared with that of the flat strain-free structure (${d}_{cc}^{0}$) and the amount of biaxial strain was calculated as *ε* = (${I}_{cc}^{{\rm{s}}{\rm{t}}{\rm{r}}{\rm{a}}{\rm{i}}{\rm{n}}{\rm{e}}{\rm{d}}}$ − ${d}_{cc}^{0}$)/${d}_{cc}^{0}$. To calculate barriers for strained flat graphene (without ripples), the in-plane positions of carbon atoms were obtained by applying biaxial strain. Extended Data Fig. 7b shows the energy barriers *E* found for the three cases.

We calculated the total energy of the proton–graphene system as a function of the position of the proton in the direction perpendicular to the centre of the hexagonal ring in the graphene crystal lattice (Extended Data Fig. 7). Our calculations showed that the proton became physisorbed at about 1 Å away from the graphene lattice, which corresponded to the minimum energy of the system. The maximum was reached when the proton was in the middle of the hexagonal ring. The barrier *E* for proton permeation is calculated by subtracting the minimum energy from the maximum one. For the case of flat unstrained graphene, the energy barrier found using these approximations is about 1.37 eV, in good agreement with the earlier theory^14^. As various approaches used to calculate *E* yield a rather large spread in the predicted values^10,11,13,14,18^ and the exact value of the energy barrier for flat monolayer graphene remains debatable^14^, here we avoid this uncertainty by focusing on relative changes in *E* that are arising from strain and curvature. Finally, note that *E* is expected to vary across membranes becoming lower around wrinkles and ripples and higher in flatter and unstrained areas. As this strain is mostly random, it is reasonable to expect that in the first approximation the distribution of *E* is normal (that is, Gaussian). As proton currents depend exponentially on *E*, their distribution should then be log-normal, which is consistent with our SECCM observations.

## Online content

Any methods, additional references, Nature Portfolio reporting summaries, source data, extended data, supplementary information, acknowledgements, peer review information; details of author contributions and competing interests; and statements of data and code availability are available at 10.1038/s41586-023-06247-6.

## Data Availability

All relevant data are available from the corresponding authors and at https://zenodo.org/record/7930090.

## References

[1] Bunch JS (2008). Impermeable atomic membranes from graphene sheets. Nano Lett..

[2] Sun PZ (2020). Limits on gas impermeability of graphene. Nature.

[3] Leenaerts O, Partoens B, Peeters FM (2008). Graphene: a perfect nanoballoon. Appl. Phys. Lett..

[4] Koenig SP, Wang L, Pellegrino J, Bunch JS (2012). Selective molecular sieving through porous graphene. Nat. Nanotechnol..

[5] Sun PZ (2021). Exponentially selective molecular sieving through angstrom pores. Nat. Commun..

[6] Mogg L (2019). Perfect proton selectivity in ion transport through two-dimensional crystals. Nat. Commun..

[7] Griffin E (2020). Proton and Li-ion permeation through graphene with eight-atom-ring defects. ACS Nano.

[8] Hu S (2014). Proton transport through one-atom-thick crystals. Nature.

[9] Lozada-Hidalgo M (2016). Sieving hydrogen isotopes through two-dimensional crystals. Science.

[10] Miao M, Nardelli MB, Wang Q, Liu Y (2013). First principles study of the permeability of graphene to hydrogen atoms. Phys. Chem. Chem. Phys..

[11] Poltavsky I, Zheng L, Mortazavi M, Tkatchenko A (2018). Quantum tunneling of thermal protons through pristine graphene. J. Chem. Phys..

[12] Mazzuca JW, Haut NK (2018). Theoretical description of quantum mechanical permeation of graphene membranes by charged hydrogen isotopes. J. Chem. Phys..

[13] Feng Y (2017). Hydrogenation facilitates proton transfer through two-dimensional honeycomb crystals. J. Phys. Chem. Lett..

[14] Kroes J, Fasolino A, Katsnelson M (2017). Density functional based simulations of proton permeation of graphene and hexagonal boron nitride. Phys. Chem. Chem. Phys..

[15] Achtyl JL (2015). Aqueous proton transfer across single-layer graphene. Nat. Commun..

[16] Walker MI, Braeuninger-Weimer P, Weatherup RS, Hofmann S, Keyser UF (2015). Measuring the proton selectivity of graphene membranes. Appl. Phys. Lett..

[17] Bentley CL, Kang M, Bukola S, Creager SE, Unwin PR (2022). High-resolution ion-flux imaging of proton transport through graphene|Nafion membranes. ACS Nano.

[18] Wang WL, Kaxiras E (2010). Graphene hydrate: theoretical prediction of a new insulating form of graphene. New J. Phys..

[19] Bartolomei M, Hernández MI, Campos-Martínez J, Hernández-Lamoneda R (2019). Graphene multi-protonation: a cooperative mechanism for proton permeation. Carbon.

[20] Huang PY (2011). Grains and grain boundaries in single-layer graphene atomic patchwork quilts. Nature.

[21] Lee GH (2013). High-strength chemical-vapor-deposited graphene and grain boundaries. Science.

[22] Gao L (2012). Repeated growth and bubbling transfer of graphene with millimetre-size single-crystal grains using platinum. Nat. Commun..

[23] Murakami, Y. *Theory of Elasticity and Stress Concentration* (Wiley, 2017).

[24] Deng S, Berry V (2016). Wrinkled, rippled and crumpled graphene: an overview of formation mechanism, electronic properties, and applications. Mater. Today.

[25] Meyer JC (2007). The structure of suspended graphene sheets. Nature.

[26] Meyer JC (2007). On the roughness of single- and bi-layer graphene membranes. Solid State Commun..

[27] Fasolino A, Los JH, Katsnelson MI (2007). Intrinsic ripples in graphene. Nat. Mater..

[28] Kosmala T (2021). Operando visualization of the hydrogen evolution reaction with atomic-scale precision at different metal–graphene interfaces. Nat. Catal..

[29] Cai J (2022). Wien effect in interfacial water dissociation through proton-permeable graphene electrodes. Nat. Commun..

[30] Mertens SFL (2016). Switching stiction and adhesion of a liquid on a solid. Nature.

[31] Kretinin AV (2014). Electronic properties of graphene encapsulated with different two-dimensional atomic crystals. Nano Lett..

[32] Gorbachev RV (2011). Hunting for monolayer boron nitride: optical and Raman signatures. Small.

[33] Ferrari AC, Basko DM (2013). Raman spectroscopy as a versatile tool for studying the properties of graphene. Nat. Nanotechnol..

[34] Chen CH (2015). Voltammetric scanning electrochemical cell microscopy: dynamic imaging of hydrazine electro-oxidation on platinum electrodes. Anal. Chem..

[35] Bentley CL (2017). Electrochemical maps and movies of the hydrogen evolution reaction on natural crystals of molybdenite (MoS_2_): basal *vs*. edge plane activity. Chem. Sci..

[36] Bentley CL, Perry D, Unwin PR (2018). Stability and placement of Ag/AgCl quasi-reference counter electrodes in confined electrochemical cells. Anal. Chem..

[37] Daviddi E (2019). Nanoscale visualization and multiscale electrochemical analysis of conductive polymer electrodes. ACS Nano.

[38] Ustarroz J, Kang M, Bullions E, Unwin PR (2017). Impact and oxidation of single silver nanoparticles at electrode surfaces: one shot versus multiple events. Chem. Sci..

[39] Wahab OJ, Kang M, Unwin PR (2020). Scanning electrochemical cell microscopy: a natural technique for single entity electrochemistry. Curr. Opin. Electrochem..

[40] Wahab OJ, Kang M, Meloni GN, Daviddi E, Unwin PR (2022). Nanoscale visualization of electrochemical activity at indium tin oxide electrodes. Anal. Chem..

[41] Ebejer N (2013). Scanning electrochemical cell microscopy: a versatile technique for nanoscale electrochemistry and functional imaging. Annu. Rev. Anal. Chem..

[42] Snowden ME (2012). Scanning electrochemical cell microscopy: theory and experiment for quantitative high resolution spatially-resolved voltammetry and simultaneous ion-conductance measurements. Anal. Chem..

[43] Xu X (2023). The new era of high-throughput nanoelectrochemistry. Anal. Chem..

[44] Mariano RG (2021). Microstructural origin of locally enhanced CO_2_ electroreduction activity on gold. Nat. Mater..

[45] Shin, Y. et al. Raman spectroscopy of highly pressurized graphene membranes. *Appl. Phys. Lett.* **108**, 221907 (2016).

[46] Zan R (2012). Scanning tunnelling microscopy of suspended graphene. Nanoscale.

[47] Edmondson, S., Frieda, K., Comrie, J. E., Onck, P. R. & Huck, W. T. S. Buckling in quasi-2D polymers. *Adv. Mater.* **18**, 724–728 (2006).

[48] Yang Q (2020). Capillary condensation under atomic-scale confinement. Nature.

[49] Cançado LG (2011). Quantifying defects in graphene via Raman spectroscopy at different excitation energies. Nano Lett..

[50] Lozada-Hidalgo M (2017). Scalable and efficient separation of hydrogen isotopes using graphene-based electrochemical pumping. Nat. Commun..

[51] Kidambi PR, Chaturvedi P, Moehring NK (2021). Subatomic species transport through atomically thin membranes: present and future applications. Science.

[52] Blochl PE (1994). Projector augmented-wave method. Phys. Rev. B.

[53] Kresse G, Furthmüller J (1996). Efficient iterative schemes for ab initio total-energy calculations using a plane-wave basis set. Phys. Rev. B.

[54] Perdew JP, Burke K, Ernzerhof M (1996). Generalized gradient approximation made simple. Phys. Rev. Lett..

[55] Grimme, S., Antony, J., Ehrlich, S. & Krieg, H. A consistent and accurate ab initio parametrization of density functional dispersion correction (DFT-D) for the 94 elements H-Pu. *J. Chem. Phys.* **132**, 154104 (2010). 10.1063/1.3382344 20423165

